# How Does Perceived Value Influence Functional Snack Consumption Intention? An Empirical Analysis Based on Generational Differences

**DOI:** 10.3390/foods14223879

**Published:** 2025-11-13

**Authors:** Xinqiang Chen, Xiu-E Zhang, Jin Yin, Jiangjie Chen, Hongyan Lin

**Affiliations:** 1 School of Economics and Management, Xiamen University of Technology, Xiamen 361024, China; chenxq23@xmut.edu.cn (X.C.); linhongyan@xmut.edu.cn (H.L.); 2 School of Business and Management, Jilin University, Changchun 130012, China; 3 College of Fine Arts, Huaqiao University, Quanzhou 362021, China; chenjiangjie@hqu.edu.cn

**Keywords:** functional snacks, perceived value, consumption intention, generational differences

## Abstract

Perceived value is a key factor shaping consumer purchase decisions. In the field of functional snack consumption, generational differences in value perception dimensions significantly influence decision-making processes, creating both challenges and opportunities for targeted marketing. Drawing on perceived value theory, this study develops a model examining the impact of perceived value on consumption intention for functional snacks. A questionnaire survey was conducted among Chinese Generation Y and Generation Z consumers, and the data were analyzed using PLS-SEM and fuzzy-set Qualitative Comparative Analysis (fsQCA). The results indicate that self-oriented values (functional and hedonic) exert a significant positive effect on other-oriented values (symbolic and social), and both categories of values positively affect consumption intention. Regarding generational moderation, the effects of functional and hedonic values on purchase intention do not differ significantly across generations; however, symbolic value has a stronger influence on Generation Z, while social value plays a more prominent role for Generation Y. Importance–Performance Map Analysis (IPMA) results further reveal differences in the relative importance and performance of value perception between the two groups. Configuration analysis shows that compared with Generation Y, Generation Z exhibits a higher threshold for strong consumption intention, a lower threshold for weak consumption intention, and greater sensitivity to value deficiency. These findings provide practical insights for functional snack companies to address generational differences and optimize marketing strategies.

## 1. Introduction

In recent years, the functional snack market has experienced explosive growth, driven by the widespread adoption of health-conscious consumption concepts and innovations in food technology. According to the 2024 China Snack Industry Blue Book, the size of China’s snack market reached RMB 1.27 trillion in 2023, with the functional food segment growing at an impressive 40%, making it the fastest-growing snack category [1]. By incorporating probiotics, plant-based proteins, vitamins, and other specific nutrients or bioactive compounds, these products retain the convenience and palatability of traditional snacks while delivering additional health benefits such as enhancing immunity, regulating gut function, and replenishing energy. Market surveys indicate that 52% of global consumers actively seek healthier food options, and nearly 30% are willing to pay a premium for health-related functions [2]. This demand continues to fuel product diversification, expanding from traditional gummies and biscuits to low-GI foods and functional dairy products. Low-GI products with glycemic management benefits and probiotic-rich items are particularly favored for their clear health advantages.

However, as the functional snack market rapidly expands, consumers’ perceptions of value have become increasingly diverse. While some groups emphasize functional attributes and sensory enjoyment, others prefer to use product choices to signal lifestyles and express self-identities [3,4,5]. This differentiation further extends to the social dimension of products and the interpretation of their symbolic meaning [6], leading to divergent consumption logics across consumer groups. Analyzing these heterogeneous value orientations helps clarify the essence of market demand and avoids homogeneous competition. In a high-growth market, such differences—if amplified—may cause firms to misallocate resources and potentially trigger consumer trust crises [7].

These divergences not only manifest at the individual level but also display clear generational characteristics. The underlying reason is that consumers from different generations have grown up in distinct technological and sociocultural environments, leading to systematic differences in values, information acquisition, and decision-making logic [8,9]. For example, Generation Y, as digital immigrants, often rely on traditional information sources such as certifications from professional institutions and product ingredient labels. In contrast, Generation Z, as digital natives, tend to form value judgments through new channels such as social media influencer reviews and user-generated content (UGC) [10,11]. Such generational differences in media use can lead to strikingly different perceptions of the same product, ultimately shaping purchase decisions.

Perceived Value Theory (PVT) provides an appropriate theoretical framework for this study. The theory posits that consumer purchase decisions are not based solely on price or single attributes but on a comprehensive evaluation of multiple values [12]. These values include functional value, hedonic value, symbolic value, and social interaction value [13,14]. Prior research has explored these dimensions in the context of functional snacks and broader food consumption. Some studies show that functional value (e.g., health benefits) and hedonic value (e.g., taste experience) significantly influence purchasing decisions [5,15]. Others reveal that symbolic and social interaction values also play crucial roles, as consumers use food choices to express individuality or integrate into social groups [16,17].

Nonetheless, existing research applying PVT has certain limitations. First, most studies focus on single-generation samples and lack generational comparisons, limiting insights into differences across age cohorts—even though generational effects are increasingly salient in consumer decision-making [18]. While prior research has acknowledged that value perceptions may differ among generations, these findings are often fragmented and descriptive rather than theoretically integrated. As a result, PVT’s explanatory scope remains constrained in capturing how consumers from different generational cohorts construct and prioritize value perceptions within diverse sociocultural contexts. Second, conventional linear analysis methods struggle to capture the complex interactions among value dimensions, which restricts a more holistic understanding of how consumers evaluate perceived value. Given that consumer evaluations are often formed through combinations of functional, hedonic, symbolic, and social value dimensions, exploring their configurational relationships across generations can deepen the theoretical understanding of perceived value and extend the application boundaries of PVT in emerging consumption markets.

On the above basis, this study proposes the core question: how does perceived value influence functional snack consumption intention? Specifically, it examines three research questions:

(1) How do different value dimensions affect consumers’ consumption intention?
(2) How do these relationships differ between Generation Y and Generation Z?
(3) Which combinations of value dimensions effectively trigger strong consumption intention?

To answer these questions, a mixed-method approach was adopted. A large-scale survey was conducted to collect data, followed by Partial Least Squares Structural Equation Modeling (PLS-SEM) to analyze causal relationships between value dimensions and purchase intention, as well as generational differences. Additionally, fsQCA was employed to identify value configurations leading to high consumption intention.

The remainder of this manuscript is structured as follows. Section 2 presents the theoretical background and research hypotheses. Section 3 explains the research methodology, including participants, instruments, and data analysis procedures. Section 4 reports the empirical results, including PLS-SEM measurement and structural model analyses, IPMA, and fsQCA findings. Section 5 discusses the implications of the results. Section 6 concludes with contributions, limitations, and directions for future research.

## 2. Literature Review and Research Hypotheses

### 2.1. Perceived Value Theory

Since Zeithaml’s seminal work, scholars have elaborated on the concept of perceived value from multiple perspectives. Zeithaml [12] defined perceived value as “the consumer’s overall assessment of a product’s utility based on the trade-off between received benefits and incurred costs.” This definition underscores the comparative and subjective nature of value perception. Later, Oliver and Holbrook [19] expanded the concept, describing perceived value as “a consumer’s preference judgment formed during interaction with a specific product,” which highlights its intrinsic subjectivity and contextual dependence. From a needs-based perspective, Sheth et al. [20] defined perceived value as “the product’s ability to satisfy multiple consumer needs.” Despite variations in wording, these definitions converge on three essential characteristics of perceived value: subjectivity and relativity, multidimensionality, and contextual dependence. In the consumption of functional foods, these traits are especially salient, as consumers weigh multiple considerations across health benefits, taste experiences, and social recognition.

The dimensionality of perceived value has developed from early unidimensional conceptualizations to a multidimensional framework. Early studies focused primarily on functional and transactional value [21]. More recent research, however, tends to treat perceived value as a multidimensional construct. For example, Leroi-Werelds [13] confirmed the mainstream acceptance of multidimensional approaches in her systematic review, while Blut et al. [22], through a meta-analysis, demonstrated that perceived value is inherently multidimensional, with each dimension exerting distinct effects on consumer evaluation. Other studies have further verified that perceived value typically includes four core dimensions: functional value, hedonic value, social interaction value, and symbolic value. As typologies have deepened, Holbrook [23] and Gallarza et al. [24] emphasized a higher-order distinction between self-oriented and other-oriented values. Self-oriented values refer to judgments derived from individual needs and preferences, such as functional utility and emotional experiences, whereas other-oriented values emphasize evaluations shaped by social reference and external recognition, such as social identity and image signaling.

Drawing on Holbrook [23] framework, this study distinguishes between self-oriented and other-oriented values in the context of functional snack consumption, and adopts four key dimensions: functional value [5], hedonic value [25], symbolic value [16], and social interaction value [26]. Functional value refers to the tangible health and nutritional benefits of a product (e.g., protein supplementation, glycemic control). Hedonic value emphasizes the sensory pleasure and emotional satisfaction of consumption (e.g., taste, enjoyment). Symbolic value captures the product’s role as an identity marker (e.g., signaling “fitness enthusiast” or “healthy lifestyle” identity). Social interaction value reflects the product’s role in facilitating interpersonal relationships (e.g., fostering community belonging, sharing behaviors). Together, these four dimensions cover both the essential characteristics of functional products and the emotional and sociocultural attributes of snack consumption, thereby providing a comprehensive lens for consumer value assessment.

### 2.2. Self-Oriented Value and Other-Oriented Value

According to Schwartz’s theory of basic human values and Holbrook’s typology of consumer value, the distinction between self-oriented and other-oriented values reflects two fundamental motivational orientations: the pursuit of personal utility and pleasure versus the pursuit of social approval and relational harmony [23,27]. Within functional snack consumption, functional value—as the core of self-oriented value—extends beyond physical health benefits. Baker et al. [28] observed that when consumers confirm a product’s nutritional and health efficacy, they develop a deeper sense of self-identity. Similarly, Applegate, Carins, Vincze, Stainer and Irwin [3] found that packaging and health-related information on protein bars reinforce consumers’ sense of self-expression.

Self-oriented values often transform into other-oriented values. Hackel et al. [29] showed that social identity significantly affects consumers’ enjoyment and sharing behaviors. This transformation process has also been empirically validated in broader consumer domains. For instance, Guo et al. [30] found that self-transcendence motives in sports consumption promote prosocial and community-oriented behaviors, while Adamczyk et al. [31] demonstrated similar value shifts from self-enhancement to social orientation in sustainable and ethical consumer choices. Folwarczny, Otterbring and Ares [16] further demonstrated that in sustainable food consumption, consumers leverage functional efficacy to manage social impressions, thereby reinforcing social interaction. Uliano, Stanco, Marotta and Nazzaro [5] found that functional and sustainable snack bars not only satisfy fitness and glycemic control needs but also act as symbols of a healthy lifestyle. Quach, Roberts, Dang, Zuo and Thaichon [26] similarly confirmed that functional and ethical attributes strengthen symbolic consumption and identity expression.

Therefore, this study proposes the following hypotheses:

**H1.**  *Functional value positively influences symbolic value.*

**H2.**  *Functional value positively influences social interaction value.*

Hedonic value, another critical self-oriented dimension, also has external effects. Cruwys et al. [32] found that pleasurable consumption experiences enhance the symbolic significance of products as group identity markers, a finding especially relevant for snack consumption. Multiple studies indicate that hedonic experiences not only fulfill immediate emotional needs but also enhance symbolic meaning and promote social interaction. For example, Hudson et al. [33] revealed that pleasurable experiences in social media contexts increase emotional satisfaction while strengthening symbolic functions. Lim et al. [34] confirmed that entertainment-based experiences in social media promote sharing and recognition, thereby enhancing social connections. Bilro et al. [35] further demonstrated that positive consumption experiences significantly increase consumers’ willingness to interact on digital platforms. Lou and Xie [36] similarly showed that consumers enjoying entertainment-driven experiences are more likely to share and interact in social networks.

Hence, the following hypotheses are proposed:

**H3.**  *Hedonic value positively influences symbolic value.*

**H4.**  *Hedonic value positively influences social interaction value.*

Close links also exist between symbolic value and social interaction value. When the symbolic meaning of a product achieves broad recognition within a social group, it becomes “social currency,” thereby stimulating interaction and connection. Schaupp and Bélanger [37] argued that brand symbolism enhances communicative value on social platforms. Gómez-Suárez et al. [38] emphasized that consumers select symbolic products to express self-concepts, thereby attracting the attention of like-minded groups. Dessart [39] showed that symbolic identification enhances relationship quality in brand interactions. Islam et al. [40] provided empirical evidence that when symbolic value aligns with group identity, consumers are more willing to share and display products in social contexts, strengthening their sense of belonging. Kumar and Kumar [41] further stressed that the formation of brand communities depends on consensus around symbolic meaning, which in turn fosters internal interaction and sharing.

The following hypothesis is proposed based on the above discussion:

**H5.**  *Symbolic value positively influences social interaction value.*

### 2.3. Perceived Value and Consumption Intention

Research has shown that self-oriented values significantly affect consumption intention. Regarding functional value, Cronin, Jr. et al. [42] found that it is positively related to customer satisfaction and recommendation intention. Annunziata and Vecchio [43], in a survey of European consumers, reported that clearly communicated nutritional benefits (e.g., cardiovascular protection, immune enhancement) significantly increase willingness to pay and repurchase behavior. Jia and Liu [44], using an extended Theory of Planned Behavior framework, found that functional value (e.g., health perceptions) significantly predicts purchase intention and enhances trust, which in turn reinforces purchase intention. Hedonic value also plays a vital role. Okazaki and Mendez [45], in a cross-cultural comparative study, revealed that consumers’ enjoyment of innovative products and technologies significantly improves adoption intention and usage persistence. Pourazad et al. [46], applying the S-O-R framework, confirmed that positive emotional experiences enhance brand attachment and emotional bonding, ultimately transforming into purchase intention.

The following hypotheses are proposed based on the above discussion:

**H6.**  *Functional value positively influences consumption intention.*

**H7.**  *Hedonic value positively influences consumption intention.*

Other-oriented values are increasingly important in digital-era consumer decision-making. Shukla and Rosendo-Rios [47] through cross-national empirical analysis, found that symbolic value strengthens purchase intention. Ortega et al. [48] in the health food context, similarly found that products perceived as lifestyle symbols remain attractive even at higher price points. Osmanova et al. [49] developed a theoretical model of symbolic value’s influence on consumption intention, confirming that brand symbolism enhances social identity and belonging, thereby driving purchase behavior. In terms of social interaction value, Liao et al. [50] found that social value (interaction, identity) in brand communities promotes participation and engagement through reciprocal norms, influencing consumption decisions. Shaheen et al. [51] examined emerging technologies and showed that AR-based social experiences significantly increase adoption intention and usage experience. Zheng et al. [52], in the health food domain, confirmed that group identity and belonging significantly strengthen purchase intention, suggesting that community involvement and belonging exert influence via social interaction value.

Therefore, this study proposes the following two hypotheses:

**H8.**  *Symbolic value positively influences consumption intention.*

**H9.**  *Social interaction value positively influences consumption intention.*

### 2.4. Generational Moderation Effects

Recent research highlights the systematic differences in value assessment and behavioral decision-making across consumer generations. Generations Y and Z, as two key consumer cohorts, differ markedly in their upbringing, technological adaptation, and consumption orientations [53,54].

Significant generational differences exist in evaluations of functional and hedonic value. Chen et al. [55] reported that Generation Y (born 1980–1994) consumers emphasize scientifically validated health efficacy when evaluating functional foods, consistent with their preference for pragmatic health solutions. By contrast, Generation Z (born 1995–2009) favors products combining functionality with sustainability. Rai et al. [56] further showed that age significantly influences the weight consumers assign to functional value. With regard to hedonic value, generational differences in sensory preferences are also evident. Compared with Generation Y, Generation Z places greater emphasis on taste, flavor, and overall sensory experience. For example, Savelli and Murmura [57] demonstrated that younger consumers’ purchase intentions are not only driven by health perceptions but also significantly shaped by sensory satisfaction. Raptou et al. [58] noted that Generation Z hesitates to adopt plant-based products when dissatisfied with taste or texture, indicating higher hedonic demands.

Hence, the following hypotheses are proposed:

**H10.**  *The effect of functional value on consumption intention differs between Generations Y and Z.*

**H11.**  *The effect of hedonic value on consumption intention differs between Generations Y and Z.*

Other-oriented values also show distinct generational patterns. Regarding symbolic value, Generation Z tends to express identity and values (e.g., sustainability, social issues) through consumption, reflecting the coupling of value orientation with media habits [59]. In contrast, Generation Y remains more sensitive to identity and status cues in certain categories [55]. When symbolic meanings align with group values, willingness to interact and share increases, thereby enhancing purchase intention [10].

For social interaction value, its influence appears particularly salient among Generation Z. Research shows that user-generated content (UGC) and micro-influencer endorsements significantly affect Generation Z’s interaction and purchase intentions [11], while AR-based “shared social experiences” enhance identity and interaction, further strengthening continuous usage and consumption [60]. Overall, symbolic value and social interaction value exert stronger effects on Generation Z, with pathways distinct from those of Generation Y.

The following hypotheses are proposed based on the above discussion:

**H12.**  *The effect of symbolic value on consumption intention differs between Generations Y and Z.*

**H13.**  *The effect of social interaction value on consumption intention differs between Generations Y and Z.*

**Figure 1:**
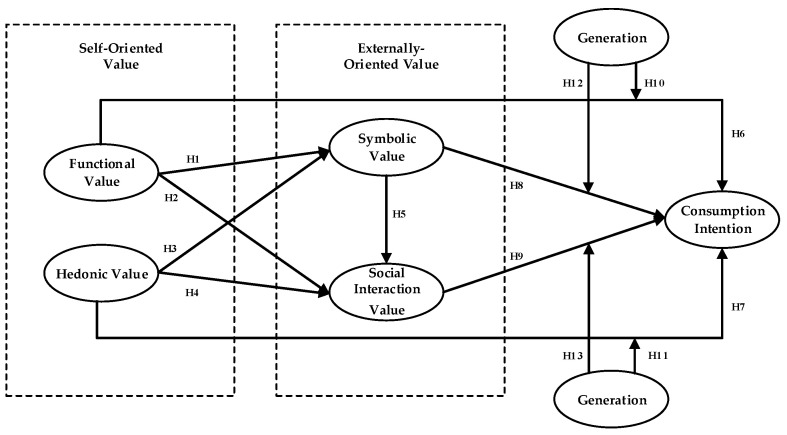
Research model.

The proposed research model and hypotheses are illustrated in Figure 1.

## 3. Research Design

### 3.1. Variable Measurement

This study draws upon authoritative literature in relevant fields to select well-established measurement scales. Industry experts were invited to review the initial questionnaire, and based on their feedback, certain items were revised or removed to form the final survey instrument. Functional value was measured by adapting items from Sweeney and Soutar [61] and Savelli and Murmura [57], focusing on the actual health and nutritional attributes of foods. Items included satisfaction of health needs and conformity with health standards. Hedonic value was measured with reference to Holbrook and Hirschman [62] scale, supplemented by Thakur, Sharma, Mehta and Torrico [4], emphasizing sensory enjoyment and immediate gratification during consumption. Symbolic value items were adapted from Wiedmann et al. [63], focusing on the personal image and lifestyle that products convey, reflecting consumers’ self-identification through product choices. Social interaction value drew on Gallarza et al. [64] multidimensional model of experiential value and Wang, Ashraf, Thongpapanl and Nguyen [60], highlighting the role of products in social contexts, such as suitability for sharing and enhancing social enjoyment. Consumption intention was measured with reference to Martins et al. [65] and Chetioui et al. [66], covering purchase planning, preference over alternatives, price acceptance, and recommendation intention, thus providing a comprehensive assessment of consumer behavioral tendencies. All items were measured on a seven-point Likert scale, with response options ranging from 1 (“strongly disagree”) to 7 (“strongly agree”). The scale items and their sources are presented in Table 1.

### 3.2. Questionnaire Design and Data Collection

The finalized items were compiled into the survey questionnaire, which consisted of three parts: (1) an introduction and instructions, (2) demographic information, and (3) the main survey items. In the survey introduction, we briefly explained that the purpose of the survey was to investigate perceived value and consumption intention in the context of functional snacks. We assured respondents of anonymity and clarified that the results would be used solely for academic research with strict protection of personal privacy. The demographic section covered gender, generation, education level, and monthly disposable income. The main body of the questionnaire focused on measuring the constructs in the theoretical model: functional value, hedonic value, symbolic value, social interaction value, and consumption intention.

Data collection was conducted online via the Tencent Questionnaire platform between 15 September and 18 October 2024. A total of 1011 questionnaires were distributed. Since the study focused on Generation Y and Generation Z consumers aged 18 and above, Generation Z was defined as those born between 1995 and 2007, while Generation Y included those born between 1980 and 1994. Screening questions were included to identify respondents’ birth years, and those outside these ranges were excluded. Additional quality control measures removed responses with excessively short completion times, uniform answers across all items, or contradictory responses. Because the survey was conducted online, potential sampling biases such as an overrepresentation of urban residents and higher-educated respondents were taken into account. To mitigate these biases, the questionnaire was distributed across different provinces and included respondents with varying educational backgrounds to achieve a more diverse sample. After screening, 807 valid questionnaires remained, yielding a response rate of 79.8%.

The demographic characteristics of respondents are shown in Table 2. Females (52.7%) slightly outnumbered males (47.3%), with a balanced distribution overall. Generation Z accounted for 55.8% of the sample, while Generation Y represented 44.2%, consistent with the generational composition of functional snack consumers. In terms of education, 44.0% held bachelor’s degrees, 7.1% held master’s degrees or higher, 29.7% had junior college diplomas, and 19.2% had secondary school or below. Regarding monthly disposable income, 38.5% reported 1000 yuan or less, 30.0% reported 1001–3000 yuan, 22.5% reported 3001–6000 yuan, and 9.0% reported above 6000 yuan. This distribution captures both low-income groups such as students and employed groups with stable income, consistent with the socioeconomic profile of Generations Y and Z.

### 3.3. Research Methods

PLS-SEM was employed as the primary analytical technique. The model consists of two components: the measurement model and the structural model. The measurement model was assessed through reliability and validity tests to ensure accuracy and consistency of the latent constructs. The structural model was evaluated using R^2^ and Q^2^ indices to examine explanatory and predictive power, and path analysis was conducted to test the 13 proposed hypotheses. To enrich the analysis, IPMA was applied to assess the relative importance of constructs. In addition, fsQCA was conducted to explore how combinations of multiple variables jointly influence consumption intention.

## 4. Results

### 4.1. Common Method Bias Tests

To mitigate potential common method bias (CMB), we took some precautions during instrument design and data collection. First, we ensured diversity and balance in item wording to avoid response tendencies caused by uniform phrasing. Second, during data collection we deliberately recruited consumers across gender, age, region, and education levels to enhance sample heterogeneity and representativeness. Further, we conducted Harman’s single-factor test on the screened data. The items automatically loaded onto four factors with eigenvalues greater than 1, and the first (unrotated) factor accounted for 48.617% of the variance, which is below the 50% threshold [67]. These results suggest that common method bias is not a serious concern in this study.

### 4.2. Assessment of Measurement Model

Table 3 reports the measurement model results, including items, factor loadings, Cronbach’s alpha (α), composite reliability (CR), and average variance extracted (AVE) for each construct.

All standardized loadings fall between 0.771 and 0.896, well above the conventional 0.50 criterion, indicating strong item–construct relationships. Cronbach’s alpha values range from 0.811 to 0.892, exceeding the 0.70 benchmark [68] and evidencing good internal consistency. Composite reliability values range from 0.874 to 0.925, all above 0.70 [69], indicating satisfactory reliability. AVE values range from 0.634 to 0.756, surpassing the 0.50 threshold [70], thus supporting convergent validity. Overall, the measurement model demonstrates strong construct validity, internal consistency, stability, and convergent validity.

We evaluated discriminant validity using two criteria. First, the HTMT ratios (Table 4) are all below 0.85 [71], indicating satisfactory discriminant validity. Second, according to the Fornell–Larcker criterion (Table 5), the square roots of AVE (diagonal bold elements) exceed the corresponding inter-construct correlations in their rows and columns [72], further supporting discriminant validity.

### 4.3. Assessment of Structural Model

Table 6 presents Q^2^, R^2^, and the Goodness of Fit (GOF). Q^2^ assesses the predictive relevance of endogenous constructs. The Q^2^ for CI is 0.563, indicating substantial predictive relevance. R^2^ reflects explained variance: SV = 0.416, SIV = 0.540, and CI = 0.659, suggesting strong explanatory power—particularly for CI [71]. The overall GOF = 0.614, exceeding the 0.36 benchmark for high fit [73], indicating good model fit.

**Figure 2:**
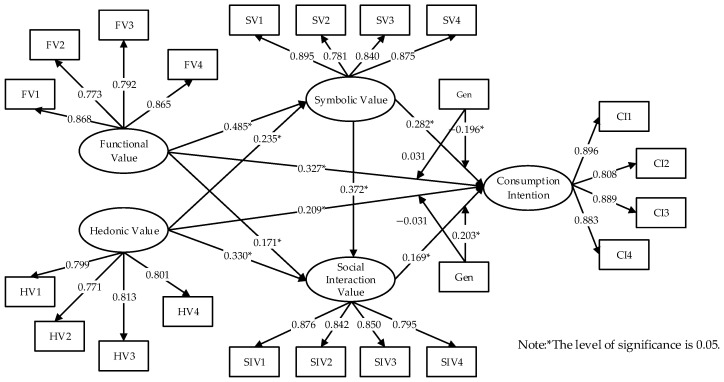
Analysis results of hypothesized model.

We tested the hypotheses using bootstrapping. Table 7 reports the path coefficients, *p*-values, VIFs, and decisions; Figure 2 visualizes the significant paths.

Self-oriented values (functional and hedonic) exert significant positive effects on other-oriented values (symbolic and social interaction): β = 0.485, *p* < 0.001; β = 0.171, *p* < 0.01; β = 0.235, *p* < 0.001; β = 0.330, *p* < 0.001, supporting H1, H2, H3, and H4. Symbolic value also significantly affects social interaction value (β = 0.372, *p* < 0.001), supporting H5.

Regarding consumption intention, both self-oriented values have significant positive effects (FV → CI: β = 0.327, *p* < 0.001; HV → CI: β = 0.209, *p* < 0.01), supporting H6 and H7. Other-oriented values also positively influence consumption intention (SV → CI: β = 0.282, *p* < 0.001; SIV → CI: β = 0.169, *p* < 0.05), supporting H8 and H9.

Generational moderation analyses show no significant differences for FV and HV effects on CI between Generations Y and Z (β = 0.031, *p* > 0.05; β = −0.066, *p* > 0.05), thus H10 and H11 are not supported. However, significant generational differences emerge for SV and SIV: the SV → CI effect is stronger for Generation Y (β = −0.196, *p* < 0.05, interaction-coded), while SIV → CI is stronger for Generation Z (β = 0.203, *p* < 0.05), supporting H12 and H13.

All path VIF values are below the conventional multicollinearity threshold (typically < 10), indicating no serious collinearity issues [74], this further indicates that common method bias is not a serious concern in this study.

### 4.4. Importance–Performance Map Analysis

IPMA is a two-dimensional approach for jointly assessing each construct’s strategic importance and its performance [75]. The horizontal axis captures relative importance (total effect on the target construct), while the vertical axis presents performance (standardized on a 0–100 scale). To examine generational differences, we ran group-specific IPMAs for Generations Y and Z and compared coordinate patterns across key value dimensions.

**Figure 3:**
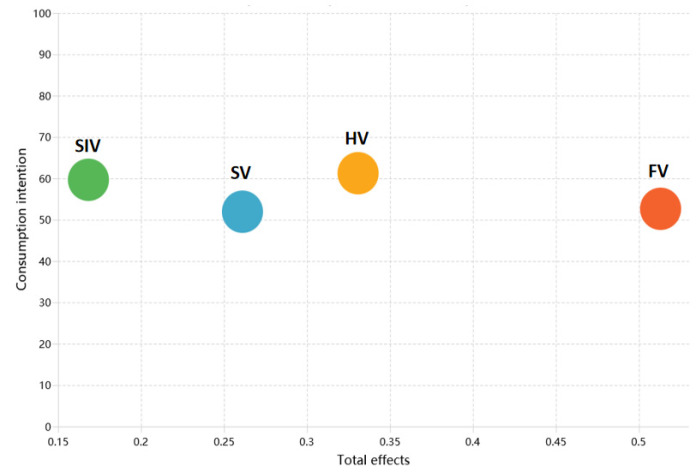
Importance–performance map analysis: Generation Y.

Figure 3 shows the IPMA for Generation Y. In terms of importance, FV exerts the strongest influence on consumption intention, followed by HV and SV, with SIV being lowest. Performance-wise, FV scores highest, HV and SV are moderate, and SIV is the lowest. These results suggest that Generation Y places greatest emphasis on product efficacy, followed by affective experience and symbolic meaning, and pays the least attention to social attributes.

**Figure 4:**
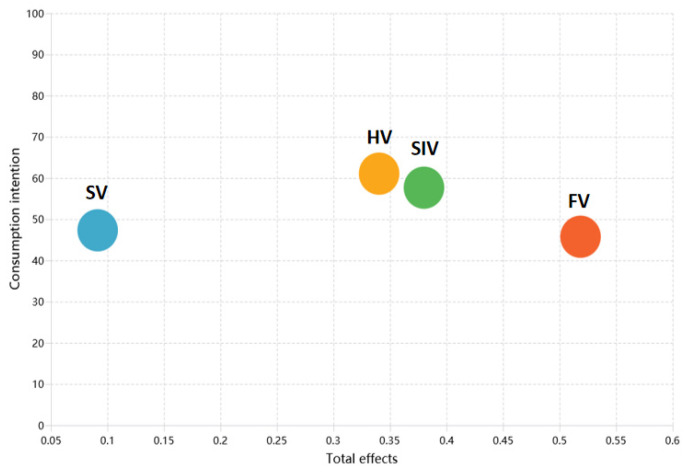
Importance–performance map analysis: Generation Z.

Figure 4 presents the IPMA for Generation Z. While FV remains the most important driver, the ordering changes notably: SIV rises to second place; HV drops one position yet shows a slight increase in importance; and SV ranks last. In terms of performance, all dimensions except HV score lower for Generation Z than for Generation Y.

### 4.5. Fuzzy-Set Qualitative Comparative Analysis

Given that purchase decisions for functional snacks arise from multifaceted, conjunctural causation, and traditional SEM has limitations in modeling complex configurations, we employed fsQCA to examine how different combinations of conditions jointly lead to the outcome. This approach enables a finer-grained understanding of causal complexity and equifinality in consumer decision-making.

Following established practice, we calibrated cases into fuzzy sets using the direct method, transforming raw data into membership scores in the [0, 1] interval. Specifically, we used the 5th, 50th, and 95th percentiles of the empirical distributions as the three anchors for full non-membership, crossover (maximum ambiguity), and full membership, respectively [76].

We then tested for necessary conditions within each generation. Table 8 and Table 9 report the necessity analyses for the Generation Y and Generation Z cohorts. All consistencies are below 0.90 [77], indicating that no single condition is necessary for the outcome. This supports the view that high consumption intention emerges from complex interactions among multiple conditions.

For configurational analysis, we followed Kraus et al. [78], setting the consistency threshold to 0.80, PRI consistency to 0.70, and minimum case frequency to 2. Results are summarized in Table 10. All solutions exhibit high consistencies between 0.897 and 0.973, exceeding the acceptable 0.750 level. Raw coverages range from 0.268 to 0.698, with most solutions above 0.500, indicating good explanatory power. Woodside [79] indicated that raw coverage above 0.200 is considered effective; all solutions meet this criterion.

For Generation Y, two configurations lead to high consumption intention. M1 indicates that the joint presence of FV, HV, and SIV yields high CI. M2 shows that FV + SV + SIV also leads to high CI; in both configurations, FV is a core condition. In the not-high CI solution for Generation Y, although all value dimensions are absent, the absence of FV appears only as a peripheral condition, suggesting its foundational role in Y’s decision process.

For Generation Z, higher thresholds are observed. The high CI configuration M1 requires the simultaneous presence of all four value dimensions (FV, HV, SV, SIV). In the not-high CI solutions, Z exhibits higher sensitivity: while a universal absence of values naturally lowers CI (M1), M2 shows that even with HV and SV present, the absence of FV and SIV is sufficient to suppress CI.

## 5. Discussion

Grounded in Perceived Value Theory, this study developed a model of consumption intention for functional snacks among Generations Y and Z, applying PLS-SEM and configurational analysis to systematically uncover the mechanisms by which value dimensions shape purchase intention and the ways in which these effects differ across generations. The results are discussed as follows.

### 5.1. The Impact Mechanisms of Perceived Value

Our findings show that self-oriented values (functional value and hedonic value) exert significant positive effects on other-oriented values (symbolic value and social interaction value). This is highly consistent with the value framework of Schwartz and Cieciuch [80], which emphasizes that individual values do not exist in isolation but form an interrelated system where the fulfillment of foundational values drives the pursuit of higher-level ones.

Among other-oriented values, symbolic value significantly influences social interaction value. Previous research has often treated symbolic value and social interaction value as separate constructs. However, our results reveal a dynamic reinforcing mechanism between the two, filling a gap left by Sheth, Newman and Gross [20] consumption value model, which paid limited attention to internal linkages among other-oriented values. Specifically, the symbolic meaning of functional snacks (e.g., signaling a healthy image or fashionable lifestyle) is frequently conveyed through social settings, while social interaction in turn strengthens individuals’ perception of symbolic value.

With regard to the impact of perceived value on purchase intention for functional snacks, the results show that both self-oriented and other-oriented values exert significant positive effects. Consumers evaluate products holistically by integrating health, nutrition, taste, and social meaning, and these multidimensional judgments directly drive purchase decisions [57]. Functional value emerges as the most influential factor, confirming that nutritional and health-related benefits remain the core motivation for functional snack consumption [58]. This pattern is consistent with international findings. Bech-Larsen and Grunert [81] observed that consumers in Denmark, Finland, and the United States commonly associate functional attributes with perceived healthiness, underscoring the universal relevance of utilitarian value. Likewise, Banna et al. [82] showed that both Chinese and American young adults link healthy eating to a balance between functionality and enjoyment, suggesting that the joint influence of functional and hedonic values reflects a shared evaluative logic across cultures.

### 5.2. Findings on Generational Moderation Effects

This study finds no significant generational differences in the effects of functional and hedonic values on purchase intention. This diverges somewhat from Savelli and Murmura [57] findings on Gen Z’s health food consumption, which emphasized younger consumers’ stronger preference for pleasure and affective experiences. Our results suggest that Generation Y also regards functionality and enjoyment as central purchasing criteria, indicating that such basic value demands transcend generational boundaries and exhibit broad commonality.

For other-oriented values, the results reveal patterns not fully consistent with prior studies. On the one hand, Generation Z’s heightened sensitivity to social interaction value aligns with Halicka et al. [83], who found that younger groups are more inclined to integrate food consumption with community interaction and sharing. On the other hand, our findings supplement Chen, Xu, Tang and Zheng [55], who reported that Generation Z emphasizes cultural and local symbolism in dietary choices. Here, we find that Generation Y also demonstrates strong symbolic needs in functional snack consumption. Such generational asymmetry may stem from differences in digital socialization and identity construction processes. Generation Z, having grown up in an environment dominated by social media and algorithmic interaction, tends to construct and negotiate their identities through online symbolic expression and peer engagement. Consequently, social interaction and symbolic cues embedded in products play a central role in reinforcing their self-concept and social belonging. In contrast, Generation Y’s identity projects are less digitally mediated, which may explain their relatively pragmatic orientation toward symbolic value. This suggests that symbolic value is not unique to younger cohorts but may redistribute across generations depending on the consumption context.

### 5.3. IPMA Findings

For Generation Y, functional value ranks highest in both importance and performance, while social interaction value ranks lowest. This reflects a pragmatic orientation toward functional snack consumption and resembles the consumption patterns of traditional cohorts described by Fathin et al. [84], where priority is given to practical efficacy, followed by affective and symbolic needs.

In contrast, Generation Z shows a markedly different ordering. Although functional value remains the most important driver, social interaction value rises to second place, while symbolic value falls to last. This finding corrects earlier one-sided assumptions about Gen Z’s value hierarchy and highlights their socially driven approach to functional snack consumption: products serve not only as tools for self-expression but also as instruments for sustaining social ties, for example, through sharing snacks and engaging in community “check-in” practices. Moreover, Generation Z’s performance scores for functional, symbolic, and social interaction values are all lower than those of Generation Y, suggesting that their expectations of functional snacks are higher. This provides a valuable direction for companies to enhance product offerings.

### 5.4. Configurational Analysis

The configurational analysis identifies multiple pathways to high purchase intention, with distinct generational differences. For Generation Y, two causal pathways emerge: M1, the simultaneous presence of functional, hedonic, and social interaction values; and M2, the co-occurrence of functional, symbolic, and social interaction values. In both cases, functional value is a core condition, indicating its indispensable role in Generation Y’s decision-making.

For Generation Z, a higher threshold is evident. High purchase intention requires the simultaneous presence of all four value dimensions (functional, hedonic, symbolic, and social interaction), revealing more comprehensive demands: products must be practical and enjoyable while also fulfilling social and self-expressive needs. Previous research, often based on linear regression, has tended to emphasize the direct effects of single value dimensions on Gen Z’s consumption behavior [85,86]. By applying configurational analysis, this study demonstrates that Gen Z’s purchase intentions arise from the joint effects of multiple values, thereby deepening understanding of the complexity of their consumption demands. In terms of low purchase intention, Generation Z displays greater sensitivity. While the complete absence of all values naturally reduces intention, even when hedonic and symbolic values are present, the absence of functional and social interaction values is sufficient to suppress purchase. This underscores Gen Z’s strong reliance on core values and suggests that their comprehensive value expectations may stem from their unique growth environment, which fosters higher and more multidimensional product expectations.

## 6. Conclusions and Implications

### 6.1. Research Conclusions

Through a series of empirical analyses, this study arrives at some conclusions. Regarding the relationships among perceived value dimensions, self-oriented values (functional value and hedonic value) significantly and positively influence other-oriented values (symbolic value and social interaction value). Both self-oriented and other-oriented values exert significant positive effects on purchase intention, with functional value showing the strongest impact. In terms of generational differences, the effects of functional and hedonic values on purchase intention do not differ significantly between Generations Y and Z. However, symbolic value has a stronger effect on Generation Z, while social interaction value exerts greater influence on Generation Y.

The IPMA further reveals that for Generation Y, functional value ranks highest in both importance and performance, while social interaction value ranks lowest. For Generation Z, functional value remains most important, but social interaction value rises to second place and symbolic value drops to last. Except for hedonic value, the performance scores of other dimensions are lower for Generation Z than for Generation Y.

Configurational analysis shows that Generation Y has two pathways to high purchase intention, both with functional value as the core condition. For Generation Z, high purchase intention requires the simultaneous presence of functional, hedonic, symbolic, and social interaction values. Moreover, Generation Z is more sensitive to value deficits: even when hedonic and symbolic values are present, the absence of functional and social interaction values suppresses purchase intention.

### 6.2. Theoretical Contributions

First, building on Perceived Value Theory, this study develops a transmission mechanism framework of “self-oriented value → other-oriented value → purchase intention,” thereby extending the theoretical boundary of PVT. Unlike prior research that often examined single value dimensions in isolation, this study demonstrates how self-oriented values (functional and hedonic) directly influence other-oriented values (symbolic and social interaction), and how these jointly shape purchase intention. By incorporating generational differences as moderating factors, the study also reveals distinct patterns across Generations Y and Z, offering theoretical support for the application of PVT in the segmented context of functional food consumption.

Second, by employing IPMA, this study goes beyond the limitations of traditional path analysis that focus solely on effect sizes. By simultaneously assessing both importance and performance, it highlights generational divergences in value priorities, providing methodological innovation for identifying consumer heterogeneity and enriching the analytical toolkit for empirical applications of PVT.

Third, from a configurational perspective, the study uncovers complex multi-value pathways leading to functional snack purchase intention. Traditional methods often emphasize net effects of single dimensions while overlooking interactions and combinatory effects. Using fsQCA, this study identifies dual pathways for Generation Y (functional + social interaction/symbolic) and a “full value fulfillment” high-threshold pattern for Generation Z, showing how different value combinations jointly shape decisions. This advances understanding of the complexity and multiplicity of consumer behavior.

### 6.3. Practical Implications

For Generation Y consumers, functional value remains the dominant driver of purchase intention, while social interaction value plays a relatively minor role. Companies should therefore emphasize function-oriented positioning by strengthening nutritional and health attributes and highlighting ingredient information, efficacy, and scientific credibility. Campaigns can focus on communicating tangible benefits, supported by professional endorsements and transparent labeling. Offline experiential activities—such as product demonstrations or workplace health programs—can further reinforce practical recognition and brand trust among this cohort.

In addition, the configurational analysis indicates that Generation Y’s purchase intention is often shaped by the joint presence of functional and either symbolic or social interaction values. Enterprises can thus design consumption scenarios that combine functionality with emotional or social meaning—for example, energy bars positioned as workplace boosters (functional + symbolic) or family sharing packs emphasizing togetherness (functional + social).

For Generation Z consumers, purchase intention depends on the simultaneous presence of multiple value dimensions—functional, hedonic, symbolic, and social interaction—reflecting broader and more integrated expectations. Although functional value remains fundamental, Generation Z also views consumption as a means of social connection and self-expression. Enterprises should therefore create socially shareable and symbolically rich products through co-branded collaborations, influencer partnerships, or youth-oriented packaging design. Online engagement initiatives such as hashtag challenges, digital communities, and user-generated content campaigns can effectively amplify social interaction value and foster emotional attachment.

Given Generation Z’s heightened sensitivity to value deficits, firms must ensure that both functional quality and social experience are consistently delivered. Neglecting these core components, even in premium or symbolically positioned products, may weaken purchase intention. Product designs that integrate health, taste, symbolism, and sociability—such as low-sugar snack boxes coupled with social media “check-in” activities—can satisfy Generation Z’s multidimensional demands and sustain brand engagement.

### 6.4. Limitations and Future Research

Despite its contributions, this study has some limitations. Methodologically, it relied mainly on survey data. While this approach enables broad coverage and efficient data collection, it may also be subject to limitations such as subjective bias in item design and the authenticity of self-reported responses. In addition, data were collected through an online survey platform, which may affect the representativeness of the sample and introduce potential biases related to respondents’ demographics or accessibility. Future studies could combine multiple methods—including field research, in-depth interviews, and consumer experiments—to more accurately capture real-world behaviors and decision-making processes across contexts, thereby deepening understanding of the formation of purchase intention.

In terms of theoretical modeling, although this study explored the effects of self-oriented and other-oriented values and revealed their complex relationships, other potentially important factors were not considered, such as cultural background, consumption habits, and health literacy. These may also significantly shape purchase intention. Future research could expand the framework by incorporating these variables to develop a more comprehensive understanding of functional snack consumption behavior.

## Figures and Tables

**Table 1:** Questionnaire items.

Construct	Items	Source
Functional Value (FV)	FV1: I believe this snack helps me meet specific health needs (e.g., digestion, immunity). FV2: The low-sugar/low-salt/low-fat features of this snack fit my healthy diet standards. FV3: I trust the functional claims of this snack because they are supported by authoritative certifications (e.g., health food labels). FV4: Compared with ordinary snacks, this product has superior health attributes.	Sweeney and Soutar [61]; Savelli and Murmura [57]
Hedonic Value (HV)	HV1: Eating this snack gives me a pleasurable sensory experience (taste, flavor, etc.). HV2: I enjoy the immediate pleasure this snack brings. HV3: The color, aroma, or texture of this snack makes me feel relaxed and satisfied. HV4: Even as an ordinary snack, I would choose it because of its good taste.	Holbrook and Hirschman [62]; Thakur, Sharma, Mehta and Torrico [4]
Symbolic Value (SV)	SV1: Eating this snack makes me feel aligned with the lifestyle I aspire to. SV2: The values embodied by this snack resonate with me. SV3: Choosing this snack makes me feel that I belong to a certain group. SV4: The style of this snack matches the personal image I want to project.	Wiedmann, Hennigs and Siebels [63]
Social Interaction Value (SIV)	SIV1: I think this snack is suitable for sharing at gatherings with friends. SIV2: Eating this snack with friends increases the fun. SIV3: I consider this snack a good choice for entertaining guests. SIV4: Sharing this snack with others enhances my social experience.	Gallarza, Gil–Saura and Holbrook [64]; Wang, Ashraf, Thongpapanl and Nguyen [60]
Consumption Intention (CI)	CI1: I plan to purchase this snack in the future. CI2: I would prioritize this snack over other similar products. CI3: I am willing to pay a higher price for this snack compared with ordinary snacks. CI4: I am willing to recommend this snack to others.	Martins, Costa, Oliveira, Gonçalves and Branco [65]; Chetioui, Benlafqih and Lebdaoui [66]

**Table 2:** Descriptive analysis of respondents (*n* = 807).

Sample	Category	Number	Percentage (%)
Sex	Male	382	47.3
	Female	425	52.7
Generation	Generation Y (1980–1994)	357	44.2
	Generation Z (1995–2007)	450	55.8
Education	Secondary vocational school, high school and below	155	19.2
	Junior college	240	29.7
	Bachelor’s degree	355	44.0
	Master’s degrees and above	57	7.1
Monthly disposable income	1000 and below	311	38.5
	1001–3000 yuan	242	30.0
	3001–6000 yuan	182	22.5
	6000 yuan and above	72	9.0

**Table 3:** Measurement model analysis results.

Constructs	Items	Loadings	α	CR	AVE
FV	FV 1	0.868	0.843	0.895	0.681
	FV 2	0.773			
	FV 3	0.792			
	FV 4	0.865			
HV	HV 1	0.799	0.811	0.874	0.634
	HV 2	0.771			
	HV 3	0.813			
	HV 4	0.801			
SV	SV 1	0.895	0.870	0.912	0.721
	SV 2	0.781			
	SV 3	0.840			
	SV 4	0.875			
SIV	SIV 1	0.876	0.862	0.906	0.708
	SIV 2	0.842			
	SIV 3	0.850			
	SIV 4	0.795			
CI	CI 1	0.896	0.892	0.925	0.756
	CI 2	0.808			
	CI 3	0.889			
	CI 4	0.883			

**Table 4:** Discriminant validity: Heterotrait–Monotrait ratio (HTMT).

	FV	HV	SV	SIV	Gen	CI
FV						
HV	0.643					
SV	0.712	0.574				
SIV	0.680	0.717	0.737			
Gen	0.145	0.049	0.100	0.063		
CI	0.804	0.706	0.729	0.791	0.070	

**Table 5:** Discriminant validity: Fornell–Larcker criterion.

	FV	HV	SV	SIV	Gen	CI
FV	**0.825**					
HV	0.553	**0.796**				
SV	0.615	0.503	**0.849**			
SIV	0.581	0.611	0.643	**0.841**		
Gen	−0.137	−0.007	−0.097	−0.044	**1**	
CI	0.702	0.623	0.645	0.695	−0.054	**0.870**

Note: Diagonal (bold) elements are the square roots of AVE.

**Table 6:** Results of structural model analysis.

Constructs	R^2^	Q^2^	GOF
SV	0.416	0.411	0.614
SIV	0.540	0.454	
CI	0.659	0.563	

**Table 7:** Results of the path analysis test.

Hypothesis	Path	Std Beta	*p*-Value	VIF	Results
H1	FV→SV	0.485	<0.001	1.439	Support
H2	FV→SIV	0.171	0.001	1.842	Support
H3	HV→SV	0.235	<0.001	1.439	Support
H4	HV→SIV	0.330	<0.001	1.534	Support
H5	SV→SIV	0.372	<0.001	1.713	Support
H6	FV→CI	0.327	<0.001	5.125	Support
H7	HV→CI	0.209	0.002	4.096	Support
H8	SV→CI	0.282	<0.001	5.022	Support
H9	SIV→CI	0.169	0.027	5.097	Support
H10	Gen × FV→CI	0.031	0.729	4.581	No Support
H11	Gen × HV→CI	−0.066	0.412	3.981	No Support
H12	Gen × SV→CI	−0.196	0.026	4.964	Support
H13	Gen × SIV→CI	0.203	0.025	4.986	Support

**Table 8:** Necessity Analysis Results for Generation Y Cohort.

Variable	High CI		Not High CI	
	Consistency	Coverage	Consistency	Coverage
FV	0.732	0.878	0.424	0.479
~FV	0.567	0.511	0.892	0.758
HV	0.769	0.840	0.528	0.543
~HV	0.581	0.567	0.844	0.775
SV	0.774	0.862	0.492	0.516
~SV	0.565	0.542	0.868	0.784
SIV	0.818	0.850	0.542	0.531
~SIV	0.549	0.560	0.847	0.814

**Table 9:** Necessity Analysis Results for Generation Z Cohort.

Variable	High CI		Not High CI	
	Consistency	Coverage	Consistency	Coverage
FV	0.866	0.764	0.492	0.504
~FV	0.438	0.426	0.770	0.870
HV	0.788	0.721	0.509	0.542
~HV	0.499	0.467	0.738	0.802
SV	0.800	0.754	0.511	0.560
~SV	0.533	0.484	0.776	0.818
SIV	0.838	0.741	0.527	0.541
~SIV	0.482	0.467	0.748	0.843

**Table 10:** Results of Configuration Analysis.

Configuration	Generation Y			Generation Z		
	High CI		Not High CI	High CI	Not High CI	
	M1	M2	M1	M1	M1	M2
FV	⬤	⬤	⨂	⬤	⊗	⊗
HV	⬤		⊗	⬤	⨂	●
SV		⬤	⊗	⬤	⨂	●
SIV	⬤	●	⊗	⬤	⊗	⊗
Consistency	0.941	0.950	0.938	0.897	0.973	0.949
Raw coverage	0.562	0.577	0.698	0.588	0.521	0.268
unique coverage	0.051	0.066	0.698	0.588	0.304	0.051
Solution coverage	0.628		0.698	0.588	0.572	
Solution consistency	0.939		0.938	0.897	0.961	

Note: Large circles indicate core conditions and small circles peripheral conditions. Black circles (“●”) indicate the “presence” of a condition, crossed-out circles (“⊗”) indicate its “negation,” and blank spaces in the solutions indicate “don’t care”.

## Data Availability

The data presented in this study are available on request from the corresponding author due to privacy.
